# Role of lipidomics in assessing the functional lipid composition in breast milk

**DOI:** 10.3389/fnut.2022.899401

**Published:** 2022-09-02

**Authors:** Moganatharsa Ganeshalingam, Samantha Enstad, Sarbattama Sen, Sukhinder Cheema, Flavia Esposito, Raymond Thomas

**Affiliations:** ^1^School of Science and the Environment/Boreal Ecosystems Research Initiative, Memorial University of Newfoundland, Corner Brook, NL, Canada; ^2^Neonatal Intensive Care Unit, Orlando Health Winne Palmer Hospital for Women and Babies, Orlando, FL, United States; ^3^Department of Pediatric Newborn Medicine, Brigham and Women’s Hospital, Boston, MA, United States; ^4^Harvard Medical School, Boston, MA, United States; ^5^Department of Biochemistry, Memorial University of Newfoundland, St. John’s, NL, Canada; ^6^Department of Mathematics, University of Bari Aldo Moro, Bari, Italy

**Keywords:** breast milk, bioinformatics, lipids, functional lipids, lipidomics

## Abstract

Breast milk is the ideal source of nutrients for infants in early life. Lipids represent 2–5% of the total breast milk composition and are a major energy source providing 50% of an infant’s energy intake. Functional lipids are an emerging class of lipids in breast milk mediating several different biological functions, health, and developmental outcome. Lipidomics is an emerging field that studies the structure and function of lipidome. It provides the ability to identify new signaling molecules, mechanisms underlying physiological activities, and possible biomarkers for early diagnosis and prognosis of diseases, thus laying the foundation for individualized, targeted, and precise nutritional management strategies. This emerging technique can be useful to study the major role of functional lipids in breast milk in several dimensions. Functional lipids are consumed with daily food intake; however, they have physiological benefits reported to reduce the risk of disease. Functional lipids are a new area of interest in lipidomics, but very little is known of the functional lipidome in human breast milk. In this review, we focus on the role of lipidomics in assessing functional lipid composition in breast milk and how lipid bioinformatics, a newly emerging branch in this field, can help to determine the mechanisms by which breast milk affects newborn health.

## Introduction

Breast milk is a complex biofluid of carbohydrates, lipids, proteins, enzymes, vitamins, and hormones. Lipids play several roles in an infant’s development and health (1). Functional lipids refer to lipids conferring known health benefits beyond basic nutritional requirements. Functional lipids include medium-chain triglycerides (MCTs), diacylglycerides (DAGs), fatty acid hydroxy fatty acids (FAHFA), conjugated linoleic, or linolenic acids (CLAs or CLNs), omega-3, omega-6, and omega-9 fatty acids, phospholipids, plasmalogens, and sphingolipids. MCTs are quickly hydrolyzed by gastrointestinal lipases and bile salts are not required for their hydrolyzation. The hydrolyzed products are absorbed and passed directly to the liver. As such, MCTs are not normally stored as body fat (2). Phospholipids play a crucial role in cell membrane integrity, and neural and brain development, as well as modulate the inflammatory responses in infants (3–6). Ceramide (Cer) and sphingomyelin are involved in various cellular functions such as proliferation, differentiation, apoptosis, and inflammation (7). In the cell membrane, they act as a platform for hormonal and growth factor receptors and cell signaling (8), as well as aid in the development of the immune system in infants (9). Plasmalogens play a role in the prevention of oxidative stress and act as anti-inflammatory agents in infants during the response to infection (10, 11). FAHFAs have been shown to possess anti-inflammatory and anti-diabetic properties (12). The word lipidome describes the whole set of lipid species in a cell or organ (13). Lipidomics is the study of the entire cellular lipidome at the molecular level (14). Multiple recent advances in mass spectrometric technology and workflows provide accurate analytical approaches for the identification and quantification of lipidome in biological samples, including functional lipid composition. The information generated can help to better understand how lipid metabolic pathways, metabolic flux, and network integration culminate from a systems biology perspective to impact human nutrition and health outcomes (15).

Although the beneficial effects of lipids in health promotion are widely studied, there is limited data on the structure, function, composition, and potential health benefits of functional lipids present in breast milk lipidome and their role in improving pediatric health outcomes. Applying lipidomics to the analysis of breast milk lipidome can aid in the understanding of the composition of functional lipids in breast milk and how they vary with maternal physiology. This can also assist in identifying possible contributions to infant development, lactational programming of transgenerational obesity, and pediatric health outcomes. To fill this gap, this review will discuss breast milk lipidome and the role of lipidomics in assessing breast milk functional lipid composition.

## Lipidomics

**FIGURE 1:**
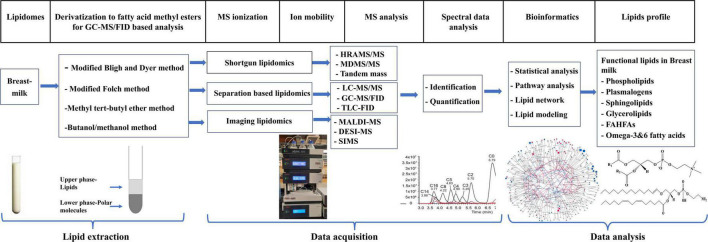
Typical workflow of lipidomic analysis of breast milk sample.

Lipidomics is a subset of metabolomics aimed at studying the metabolism, structure, and function of lipids and their roles in modulating health and disease outcome. This field has the potential to identify biomarkers for diagnosis, prognosis, prevention, and therapeutic response for various diseases (16). Innovations in mass spectrometry technology have facilitated the advancement of lipidomics as a subdiscipline in omics over recent years. The typical workflow of lipidomics consists of three major processes: sample preparation and extraction; mass spectrometry data acquisition; and data processing and interpretation (16). A summary of these processes in the context of breast milk is illustrated in Figure 1.

### Sample preparation

#### Sampling

Human breast milk studies have used several sampling protocols such as full expression, expressed alternate breast, mid-feed sampling, and fore and hind sampling. Full expression is a collection of whole milk from the breast. Expressed alternate breast refers to the collection of milk from the opposite breast of infant feeding. Mid-feed sampling is the collection of milk after 3–5 min of feeding. Foremilk sampling is the collection of milk prior to the infant feeding and hind milk is the sample right before the infant completes feeding. Furthermore, breast milk can be collected either manually or *via* electric pump (17, 18) and the fat content in the collected milk can vary during a feed. Foremilk sample contains lower fat compared to the mid and hindmilk samples (19). Fat content in milk varies from morning to evening as well. Fat content is high in evening milk compared to samples collected in the morning (20). Fat content also increases from colostrum to mature milk (21). The fat percentage of milk varies with time, feed, lactation stage, and sampling methods. The ideal method of sampling is milk collection from full expression of both breasts over 24 h with a sub-sample used for analysis. However, this is not often feasible (17, 22). Variability in milk fat with different sampling techniques necessitates those studies of human milk lipids standardize collection techniques within a study, balancing scientific rigor with practical limitations in milk collection for a given population.

#### Storage

Proper storage is critical for preventing lipolysis activity by breast milk lipase. Immediate analysis can prevent compositional changes caused by lipase activity; however, this is not always possible. To prevent lipase activity, milk samples should be stored in the freezer at –20°C for 8 days or less or –80°C for longer storage (23, 24).

### Lipid extraction methods

Modified Bligh and Dyer, Folch, methyl tert-butyl ether (MTBE), and butanol/methanol (BUME, 3:1, v/v) are the most popular extraction techniques generally used in breast milk lipidomics (25–27). Each of these techniques has pros and cons for lipid extraction and are described briefly below.

#### Modified Bligh and Dyer method

Chloroform/methanol/water (1:1:0.9, V/V/V), followed by a water wash is used to extract lipids from the whole breast milk sample. After phase separation the chloroform phase contains the total lipids. This is a widely practiced method and is very effective in extracting large amounts of lipids from the sample. The drawback of this method is the use of a hazardous solvent (chloroform) and chloroform may carry water-soluble contents during the extraction of breast milk (25, 26).

#### Modified Folch method

A total of 20 mL chloroform and methanol (chloroform: methanol 2:1) were used to extract lipids from 1 mL of breast milk (28). Then, 0.2 equivalents (of the total volume of the chloroform/methanol mixture) of water are added to the suspension to induce phase separation. It has similar advantages and disadvantages as the Modified Bligh and Dyer method (25, 27). Furthermore, This approach was unable to extract low-concentration breast milk lipids, particularly glycerol phospholipids and sphingolipids (29).

#### Methyl tert-butyl ether method

Methyl tert-butyl ether (MTBE)/methanol/water can be used to efficiently extract lipids from breast milk sample. This method does not contain a hazardous solvent such as chloroform. After phase separation, the MTBE is in the upper layer. It is used in high productivity automated lipidomics applications. The disadvantages of this method are the MTBE phase carry a significant amount of water-soluble components during extraction and MTBE is an expensive solvent (25, 30).

#### Butanol/methanol (BUME, 3:1, v/v) method

In the butanol/methanol (3:1, V/V) method, an equal volume of heptane/ethyl acetate (3:1, V/V) and 1% acetic acid is used to extract lipids from the sample. The 1% acetic acid is used to induce phase separation. This method does not carry any water-soluble components with it. The limitation of this method is that the evaporation of butanol is very difficult and limit high throughput applications (31).

#### Recommended lipid extraction methods for breast milk

The Bligh and Dyer and the Folch method are similar extraction techniques. However, the Bligh and Dyer method is a less time-consuming method and it give great lipid recovery. The MTBE method reduces cross-contamination compared to the other two methods. We recommend the Modified Bligh and Dyer method for breast milk extraction because of its simplicity, superior lipid yield, and capacity for high throughput sample extraction.

## Mass-spectrometry-based lipidomic approaches

Following lipid extraction, shotgun lipidomics, separation-based lipidomics, and imaging or spatial lipidomics are three mass spectrometry-based approaches commonly used to assess the lipidome in biological samples and can be applied to breast milk.

### Shotgun lipidomics

Shotgun lipidomics is also known as direct infusion-based lipidomics since there is no chromatographic separation performed before the sample is injected in the mass spectrometer. The lipids are ionized by a source, typically an electrospray source, to produce negative or positive ions representative of the lipids in the sample. The lipid extraction is completed by several extraction strategies based on stability, hydrophobicity, and reactivity of the lipid classes and subclasses (32). Pre-step approaches, such as derivatization, pre-separation methods, or ionization sources [such as electro spray ionization (ESI), atmospheric pressure chemical ionization (APCI), or atmospheric pressure photoionization (APPI)] are used to ionize the different lipid molecular species depending on the difficulty associated with ionization (33). The ionization of lipids is based on selective ionization. Typically, different lipid classes have different charges due to their chemical structure or composition. This is referred to as intra-source separation. For example, polar lipids are separated based on their polar head group (34). There are three approaches used in shotgun lipidomics and include tandem mass spectrometry-based shotgun lipidomics, high mass accuracy-based shotgun lipidomics, and multi-dimensional-based shotgun lipidomics (32). Shotgun lipidomics is commonly used in the lipid characterization of breast milk, especially phospholipids and Regio isomeric TAG (35, 36).

#### Tandem mass spectrometry-based shotgun lipidomics

Tandem mass spectrometry is known as MS/MS and is performed complimentary with full scan. In this approach, lipid molecules are separated according to distinct structure and class characteristics using precursor ions and specific neutral loss scans giving fragments diagnostic for example to identify the head groups in different classes of polar or membrane lipids (16).

This approach includes several advantages such as simplicity, high sensitivity, easy operational handling, and low-cost instrumentation (32). However, the selection of internal standards is difficult. Differential fragmentation kinetics of individual molecular species can influence the accuracy of the quantification and structural integrities (37, 38). Tandem mass based approaches are very effective in identifying compositional and structural features of milk lipids, especially glycerophospholipids and TAG molecular species or regioisomers (36).

#### High mass accuracy-based shotgun lipidomics

High mass accuracy can be used in quantitative and qualitative lipid analysis. This technique measures the ratio between mass and charge of fragment ions. This approach has several benefits, such as high efficiency, multifaceted, and precise measurements of lipid molecular species (32).

#### Multi-dimensional mass spectrometry-based shotgun lipidomics

Multi-dimensional mass spectrometry-based shotgun lipidomics (MDMS-SL) combines a full mass scan and all tandem mass spectrometric scans. A two-step quantification procedure is used in MDMS-SL to identify the lipid molecular species (39). Identification of individual lipid species is done by using characteristic structural units or fragments that is diagnostic of each lipid molecules (40). A two-dimensional mass map can be obtained in MDMS-SL (41). By using this approach, the lipidome from a small number of samples can be analyzed (33, 39). This method assists with identifying the individual lipid molecular species and their isomers. MDMS-SL needs less width factor compared to liquid chromatography-mass spectrometry-based lipidomics (39). Precise identification and quantification of a whole set of lipids can be obtained by this method.

### Separation based lipidomics

#### Liquid chromatography-mass spectrometry-based lipidomics

Liquid chromatography-mass spectrometry (LC-MS) is a highly effective lipidomics analysis platform that simplifies the analytical protocol and increases chromatographic segregation power and precision of detection. It can detect several lipids in a very short time (42). It is typically used in breast milk lipidomics for the identification of triglycerides and phospholipids (43). Selected and multiple reaction monitoring (SRM/MRM) are two strong quantitative methods in LCMS-based targeted analysis. These methods play a crucial role in clinical lipidomic analysis and identification of untargeted lipids (44). Data-based analysis is one of the unique procedures in LC-MS used for the identification and classification of individual lipid species by selecting most of the ions in a peak width after pre-separation. Higher peaks for untargeted analysis can be obtained in two-dimensional liquid chromatography (2D-LC) technology (45). There are two types of 2D-LC methods, namely: Reversed-phase LC (RPLC) and Normal-phase LC (NPLC). The separation of lipids using RPLC method is based on their hydrophobicity, chain length, and degree of unsaturation of fatty acyl chains. Lipid species are eluted in the following order: lipids containing longer acyl chains followed by shorter chain lipids, saturated, and polyunsaturated species. In NPLC, lipid species are identified according to their hydrophilic functionalities and their characteristic head-groups into distinct classes (15). The combination of RPLC and NPLC is the most superior separation technique in 2D-LC methods (32). RPLC classically used nonpolar C18 columns with polar solvents. Nowadays, C30 stationary phase is also used to conduct untargeted lipidomics in biological samples (46, 47).

C30 reverse phase chromatography is superior to C18 reverse phase chromatography for identifying geometric lipid isomers (48, 49). The optimized C30 reverse phase chromatography allows for excellent intra-class separation of lipid isomers with different fatty acid compositions or head group modifications. Chromatography coupled with high- resolution tandem mass spectrometry can efficiently identify lipid isomers. As a result, it can differentiate di/triglycerides, plasmalogens, and ether iso-forms of lipids in a biological sample (50).

Hydrophilic interaction liquid chromatography (HILIC) is another lipid separation technique in LC -based mass spectrometry analysis. The combination of columns with a polar stationary phase and reverse phase solvents (acetonitrile and water) is used in HILIC. Lipid separation occurs at the water/silica interface. HILIC separation is based on polar head groups, therefore they separate lipids into distinct classes based on head group composition (51, 52). Phospholipids are also identified *via* LC-MS. However, for the quantification of phospholipid molecular species, a pure standard is necessary (53). The characterization and quantification of phospholipids and sphingomyelins in breast milk is generally accomplished by using a high-performance liquid chromatography (HPLC) coupled with mass spectrometer detector. UHPLC system coupled with a high-resolution mass spectrometer are used in the identification of triglycerides and fatty acids in breast milk (54).

#### Other separation methods

Other separation-based approaches for lipid identification include gas chromatography (GC) and thin layer chromatography (TLC). GC offers a high separation, resolution, and quantification accuracy. It is suitable for lipids with high volatility and good thermal stability (32). In breast milk lipidomics research, GC with flame ionization detection (GC-FID) and GC coupled to MS (GC–MS) are commonly employed to identify total fat and fatty acid content (55–57). Due to the low volatility of phospholipids in breast milk, GC-MS cannot be used to identify it (58). Non-volatile compounds are analyzed using TLC. It is simple to use, but it lacks the separation resolution of other mass-based approaches (32). It is commonly used to identify short and long chain fatty acids, however, it is rarely used in breast milk lipidomics studies (59).

### Imaging mass spectrometry

Imaging mass spectrometry (IMS) is a new lipid imaging method which is utilized to identify the spatial distribution of lipids in a sample using two-dimensional MS technique. Several mass spectrometric-based imaging techniques are available and are differentiated based on whether they operate under vacuum or at atmospheric conditions with or without the use of a matrix. IMS has different types, such as matrix-assisted laser desorption/ionization-mass spectrometry (MALDI-MS), desorption electrospray ionization mass spectrometry (DESI-MS), secondary ion mass spectrometry (SIMS), and laser ablation secondary ion mass spectrometry (LASI-MS) (60, 61). Though imaging mass spectrometry is seldomly used in breast milk lipidomics; it has the capacity to analyze the lipid droplets or milk fat globule in breast milk especially when using applications with high spatial resolution.

#### Matrix-assisted laser desorption/ionization-mass spectrometry imaging

MALDI-IMS is used to identify lipid molecules with high-resolution imaging (62, 63). The mass to charge ratios of ionized forms of molecules are used in the identification. This process involves three major steps. First, the ionizing source changes the form of molecules into ions. Next, the mass analyzer separates the ions based on mass to charge ratio. Then, the ion detector detects and separates the ions based on their relative abundance. This is a suitable technique to visualize lipid distribution and concentration in a sample (64). This technique can assist with identifying the localization of known and unknown molecules without labeling and helps to identify lipid biomarkers based on spatial location (65).

#### Desorption electrospray ionization imaging

DESI imaging is a commonly used atmospheric pressure-based MS imaging technique (66). In DESI, charged primary organic molecules in a solution touch the top of the sample surface with definite kinetic energy. This causes the secondary molecules to move along the surface due to the action of the primary droplets. The samples are split into nanoparticles and become charged. The charged ion then enters the mass spectrometer after the charge transfer process which moves through an electric field (67). DESI assesses the inner lipid structure of biomolecules and is the best spectrometry for the surface analysis of samples. This technique does not require a matrix and is performed under atmospheric conditions (68, 69).

#### Secondary ion mass spectrometry imaging

SIMS imaging is carried out *via* the passage of a high-energy ion beam along the surface of a sample. The high-energy beam hits on the outer part of the sample producing a secondary ion. The resolution level of SIMS imaging is equal to the resolution level of optical microscopes. This technique has been applied in single-cell lipid imaging. The challenge is that it is not a soft ionization technique and lipids suffer from fragmentation using this method. The advantage of SIMS is that its ultrahigh resolution, produces three-dimensional images by separating the sample into layers at the nanometer scale (70).

### Recommended chromatographic and mass spectrometry method for breast milk lipidomics

We recommend a combination of HILIC in combination with C30 reverse phase chromatography for the routine analysis of breast milk as a complex lipid mixture. HILIC distinguishes lipids into classes including subclasses with modified head groups according to their polarity and electrostatic interactions to improve ionization and reproducibility by combining the best properties of normal phase liquid chromatography (NPLC) and reverse phase liquid chromatography (RPLC). RPLC mobile phase has high ionization efficiency compared to NPLC. HILIC/RPLC separates lipids based on the interaction between the hydrophobic stationary phase and the hydrophobicity of the fatty acyl chains. It allows for differentiation of lipid molecules based on chain length and degree of unsaturation, allowing for intra-class separation of lipids (50–52). C18 reverse phase (RP) is a widely used non-polar stationary phase used in lipidomics (49). Recently, C30 RP has been gaining popularity in lipidomic analysis due to higher peak shape selectivity permitting superior resolution of geometric lipid isomers. C30 RP is efficient in identifying lipid isomers with different fatty acid compositions, degrees of unsaturation, or head group modifications. Furthermore, C30 chromatography was shown to be very good at distinguishing and resolving triglyceride isomers which only differed based on the alternation of the fatty acids at their sn1, sn2, and sn3 positions. Considering triglycerides account for the majority of the breast milk lipidome, C30 RP chromatography would be indispensable in determining novel triglyceride regioisomers during routine analysis. Formate buffer is used in the C30 RP chromatography, while acetate buffer is used in the HILIC solvent systems. This differentiates lipids separated using HILIC as acetate adducts and those by C30 RP as formate adducts when combined with mass spectrometry. HILIC can resolve breast milk lipids into different classes including subclasses with modified head groups. On the other hand, C30 RP chromatography allows the intra-class separation of breast milk lipid isomers based on chain length and degree of unsaturation. We recommend combining HILIC and C30 RP coupled with high resolution accurate mass tandem mass spectrometry as the best approach to identify neutral lipids, modified lipids, di/triglycerides, plasmalogens, and ether iso-forms of lipids as well as isobars and regioisomers (*sn*-positional isomers) during routine lipid analysis of breast milk (50). The breast milk lipidome is complex and comprised of different classes of lipids such as phospholipids, sphingolipids, plasmalogens, triglycerides, glycolipids, and modified lipids. High resolution accurate mass tandem mass spectrometry will allow the resolution of lipids based on their molecular weight down to 3–5 ppm. Combining the resolution provided by HILIC and C30 RP chromatography with the high resolving mass power of high-resolution accurate mass tandem mass spectrometry provide superior capabilities in resolving breast milk lipids according to classes, modification in head group or fatty acids, isobars, linkages between monomers, and isomeric composition during routine lipidomics.

HILIC complements with C30 RP coupled with high resolution accurate mass tandem mass spectrometry can be very effective in resolving breast milk lipidome during targeted and untargeted analysis and is recommended as one of the best approaches to consider during routine analysis of breast milk.

## Bioinformatics for lipidomics and assessing functional lipids in breast milk lipidome

Bioinformatics is a new emerging discipline combining computer science, mathematics, physics, and biology to help with the management of data in modern biology (71). Large amounts of lipidomics data generated from breast milk lipidome can be derived from mass spectrometric analysis and the statistical analysis. Identifying methods to best evaluate the data can be challenging. Bioinformatics includes advanced databases, computational, and statistical techniques supported by mathematical theory to solve formal and practical problems in the analysis of the data. Bioinformatics in lipidomics is the combination of pre-processing and analyzing data sets using both univariate and multivariate approaches such as lipid modeling, correlation tests, dimensionality reduction, clustering or ordination techniques, classification, pathway, and network analysis (72).

### Data pre-processing for functional lipid identification and quantification in breast milk

Data processing helps to convert the mass spectrometry data from biological samples (breast milk) into a final lipidomics (breast milk lipidomics) dataset. The final lipidomics data can be used for both quantitative and qualitative analysis as well as data interpretation (73–75).

#### Lipid databases and identification tools

LIPID MAPS is a database used to assist in identifying possible structures from mass spectrometry data. It has several tools such as structural classification and nomenclature, statistical tools, computer-aided spectral comparisons, and MS/MS fragment information (32). Lipid finder, Lipid QA, Lipid miner, and Lipid search are widely used tools in lipid identification. They use the fragment information generated by mass spectrometry for the identification and quantification of lipid species (32).

Metabolomics data processing approaches can also be applied in lipidomics mass spectrometry-based applications. MZmine is one of the open-source software widely used (73). This is a Java-based software package comprising multiple data processing steps, such as spectral filtering, peak detection, alignment, normalization, Exploratory Data Analysis (EDA), and visualization. MZmine is flexible to integrate new algorithms and applications, and thus encourages active community participation in new algorithm and application development within its framework. It can also be parallelized, which makes it suitable for large-scale profiling applications such as in large clinical studies (76).

### Statistical data analysis

Statistical analysis is a very important aspect of the analytical workflow due to the high dimensionality typical of breast milk lipidomics dataset (77). This workflow ranges from univariate to multivariate approaches depending on the structure of the data and is aimed primarily at reducing the dimensionality of the data, as well as identifying features in the data that will facilitate meaningful interpretation of trends in the data of biological relevance or significance.

#### Univariate analysis

These are a collection of statistical approaches used to analyze the data based on the research hypothesis. Univariate analysis, the basic form of statistical analysis, is a recognized hypothesis testing approach in data analytics and it is often used to compare the mean of groups. For the comparison of two or more groups, one may resort to univariate methods such as the *t*-test, the Wilcoxon and Mann-Whitney test, or the analysis of variance (ANOVA) depending on the distribution of data (78). This kind of analysis is used to inspect one variable at a time in lipidomics and other fields (79). Univariate tests can be applied to all types of variables and outcomes and their results are easy to interpret. A disadvantage of univariate tests is that they disregard relations between features that may be important for biological interpretation, or which may improve statistical inference through their inclusion in the statistical test (80). Furthermore, this approach can be challenging to interpret in large dimensionality data sets.

#### Multivariate analysis

Multivariate analysis plays an important role in the extraction of valuable information from large dimensionality data such as the mass spectral data obtained following breast milk lipidomics. The advances in mass spectrometry increase the quantity and dimensionality of raw lipid data (81). For analysis, the high dimension data needs to be changed to more meaningful lower-dimensional data without losing important information. The selection of the most appropriate techniques to reduce the dimensionality of the data is one of the challenging factors in multivariate analysis. Principal Component Analysis (PCA), Canonical Correlation Analysis (CCA), Independent Component Analysis (ICA), and Partial Least-Squares Discriminant Analysis (PLS-DA), factor analysis are some dimensionality reduction techniques widely used (82). Most of these approaches are unsupervised method that aim to reduce the size of the original data projecting it in a new low-dimensional space by some assumptions. PCA is one of the most widely used method and it reduces the original data space by constructing new orthogonal dimensions in which the original data can be projected (83). These orthogonal variables are called principal components, and are linear combination of original variables that explain the maximum variance of the data (84). CCA is another multivariate approach exploiting the correlation between variables. Differently from PCA, it finds components maximizing the amount of correlation in the data (85). PLS-DA is a supervised method widely used in the analysis of high-dimensional lipidomic data. It increases the covariance rather than the correlation between components following some prior information already available from data (85). Multivariate techniques help to identify unique features in lipidomic data sets. These can facilitate useful interpretation of the data (such as biomarkers discovery or lipid metabolism associated with disease phenotypes) and may simplify the interpretation of complex biological models or data.

### Lipid pathway and network analysis

Pathway and network analysis require a similar approach with some differences. Networks are large-scale systems which are generated from omics data whereas lipid pathways are small scale-systems that are constructed from literature (86). Lipid pathway analysis is currently a major analytical tool in lipidomics. This is based on biological databases with statistical testing, mathematical analyses, and computational algorithms (87). The interactions between compounds in lipid pathway analysis help to identify biomarkers of diseases, molecular mechanisms, and biological activity that may reflect known biological pathways (86). Organizing biological processes into useful, interactive pathways and networks is the biggest challenge in bioinformatics. SphinGOMAP and LIPID MAPS Biopathways Workbench (LMBP) are some of the lipid pathway software available (88, 89). The lipid network is another emerging tool in lipidomic data analysis. Biological (Lipid) networks are graphical and mathematical tools used to describe complex biological systems (90). Relationships/interactions and variables are noted by edges and nodes, respectively. Networks show the magnitude (node size), the directionality of changes (node fill or border colors), and lipid class (node shape). Significant changes in lipids are denoted by node colors. Nodes for species failing to reach statistical significance are denoted with colored borders, and non-measured or undetected species are displayed in a different color (91). Meta Map R is a free software that helps to merge molecular biochemical and structural information with mass spectral similarity based on correlations (92, 93). This allows for a clear display of the relationship between lipidome and clinical outcomes of infants.

All these methods from multivariate and univariate analysis, such as PLS-DA, PCA, and ANOVA, are commonly used in breast milk lipidomic studies (55, 94). However, other approaches in this fields commonly used in lipidomic research can also be applied in human breast milk lipidomic studies. This will assist in the identification of novel lipid biomarkers and the construction of biological models or metabolic pathways to better understand breast milk lipid metabolism and relationships to health outcomes from a systems biology perspective.

## Lipids in breast milk

Human milk is a complex biofluid of macronutrients consisting of proteins, lipids, carbohydrates, vitamins, minerals, and trace elements. The composition of breast milk varies with several factors, such as stage of lactation, environmental factors, maternal age, body composition, and diet of a lactating mother. Breast milk has several long-term and short-term health benefits for breastfed infants (95, 96), such as adequate growth, cognitive development, immune development, regulation of inflammation and infection, and reduction of metabolic and cardiovascular disease in adult life (55, 97).

**FIGURE 2:**
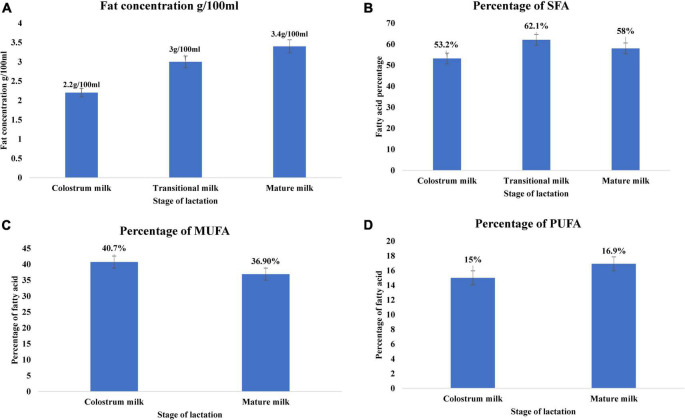
Lipid profile of breast milk across lactation stage (21). **(A)** Fat concentration (g/100 mL) of breast milk across the lactation stage. **(B)** Percentage of saturated fatty acid of breast milk across lactation stage. **(C)** Percentage of monounsaturated fatty acid of breast milk across lactation stage. **(D)** Percentage of polyunsaturated fatty acid of breast milk across lactation stage. Lactation stage—colostrum milk 0–5 days of postnatal life, transitional milk—6–15 days of postnatal life, and mature milk—16–60 days of postnatal life.

**FIGURE 3:**
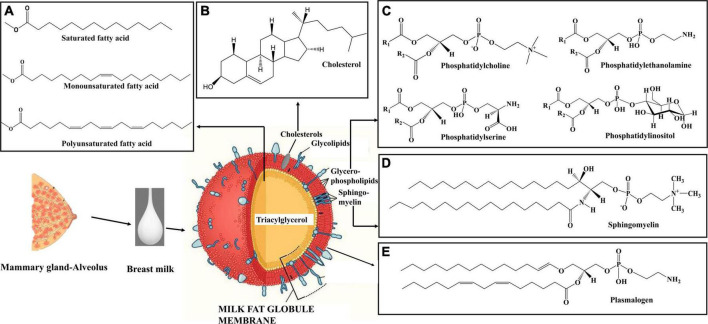
Schematic diagram of milk fat globule (108). **(A)** Structure of different types of fatty acids in breast milk triacylglycerol. **(B)** Structure of cholesterol in breast milk. **(C)** Structure of phospholipid classes in breast milk. **(D)** Structure of sphingomyelin in breast milk. **(E)** Structure of plasmalogen in breast milk. The milk fat globule membrane structure was modified from Mead Johnson Nutrition (108). Structures were created using Chem Draw 16.

Among the macronutrients present in breast milk, lipids are a crucial source of energy for neonates up to 6 months of age (55, 98). Infants gain approximately 50–60% of energy from lipids. The fat content of breast milk varies significantly among colostrum, transitional milk, and mature milk, increasing with lactation stage (99, 100). Colostrum fat content is approximately 2.2 g/100 ml, increasing to 3 g/100 ml in transition milk, and 3.4 g/100 ml in mature milk (Figure 2A) (21). Lipids in breast milk exist structurally as fat globules (Figure 3) consisting mainly of triglycerides encircled by a double layer milk fat globule membrane (MFGM), which is composed of phospholipids, sphingolipids, protein, and cholesterol (98). The inner membrane consists of phosphatidylethanolamine as a single layer and the outer membrane is made of phosphatidylcholine, sphingomyelin, cholesterol, glycoproteins, bioactive peptides, cerebrosides, and gangliosides (55, 98).

### Lipid composition of breast milk

**FIGURE 4:**
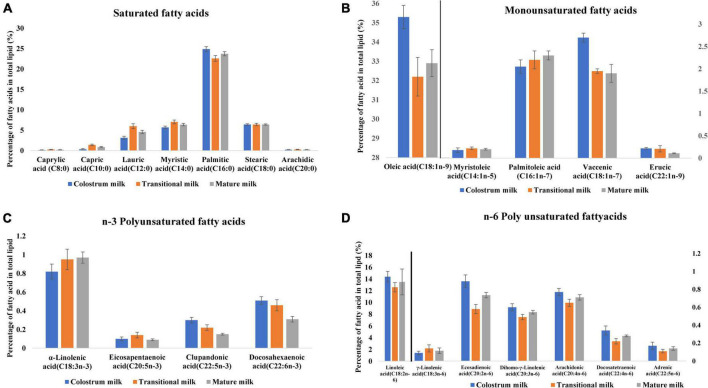
Fatty acid profile of breast milk across lactation stage (21). **(A)** Saturated fatty acids profile of breast milk across lactation stage. **(B)** Monounsaturated fatty acids profile of breast milk across lactation stage. **(C)** *n*-3 Polyunsaturated fatty acids profile of breast milk across lactation stage. **(D)** *n*-6 Polyunsaturated fatty acids profile of breast milk across lactation stage. Lactation stage—colostrum milk 0–5 days of postnatal life, transitional milk—6–15 days of postnatal life, and mature milk—16–60 days of postnatal life.

Milk fat globules are secreted by mammary alveolar cells (Figure 3). Triglycerides, comprising 98–99% of milk lipids, and a small number of monoglycerides and diglycerides are in the center of the milk fat globule forming a lipid rich core (Figure 3A). The glycolipids in the core are encircled by a milk fat membrane comprised of esterified cholesterol (Figure 3B), phospholipids (Figure 3C), sphingolipids (Figure 3D), glycosylated polypeptides, filaments, mucin, and other components (53, 101). Triglycerides contain three fatty acids (99), which can be divided into saturated fatty acids (SFA), monounsaturated fatty acids (MUFA), and polyunsaturated fatty acids (PUFA). SFAs are abundant in human milk (102), followed by MUFAs, and PUFAs. SFAs in human milk ranges from 53.2 to 58% of total fatty acid, MUFAs from 23 to 55%, and PUFAs from 6 to 36% (98). SFAs in breast milk are caprylic acid (C8:0), capric acid (C10:0), lauric acid (C12:0), myristic acid (C14:0), palmitic acid (C16:0), stearic acid (C18:0), and arachidic acid (C20:0) (103). Total SFA content increases from colostrum to transitional milk and decreases in mature milk (Figure 2B) (98). Palmitic acid is the most abundant SFA in breast milk and decreases significantly from colostrum (23.2%) to mature milk (19.8%) (Figure 4A) (21). MUFAs in breast milk are myristoleic acid (C14:1 n-5), palmitoleic acid (C16:1 n-7), oleic acid (C18:1 n-9), vaccenic acid (C18:1 n-7) and erucic acid (C22:1 n-9). MUFAs decrease from 40.7% in colostrum to 36.9% in mature milk (Figure 2C) (55). Oleic acid is the most abundant MUFA in breast milk and ranges from 35.3% in colostrum to 32.9% in mature milk (Figure 4B) (21). Total PUFAs increase from 15% in colostrum to 16.9% in mature milk (Figure 2D) (55). PUFAs, such as linoleic acid (LA, C18:2 n-6) and α-linolenic acid (ALA, C18:3 n-3), are precursors of long chain PUFAs, such as arachidonic acid (AA, C20:4 n-6), eicosapentaenoic acid (EPA, C20:5 n-3) and docosahexaenoic acid (DHA, C22:6 n-3). LA and ALA are the abundant PUFAs in breast milk and increase with lactation stage (Figures 4C,D) (21).

### Complex lipids in breast milk

In breast milk, 0.2–2% of total lipids (approximately 10–40 mg/100 mL) are complex lipids (104, 105). Complex lipids are rich in MFGMs and extracellular vesicles in breast milk (21). Complex lipids impact the physiology of the brain, gut, and skin through signal transmission and cell recognition (104). Glycerophospholipids are phosphorous containing polar lipids with different head groups in the sn-3 position. Phosphatidylinositol (PtdIns), phosphatidylethanolamine (PtdEtn), phosphatidylserine (PtdSer), and phosphatidylcholine (PtdCho) are the classes of phospholipids in human milk which have inositol, ethanolamine, serine, and choline head groups, respectively (55). The sphingolipid metabolic pathways are complex and are metabolically interrelated with each other by an enzymatic breakdown. Through this complex reaction they form ceramide, sphingomyelin, and glycosphingolipids in human breast milk (106). Sphingolipids are formed by a sphingoid base which is connected with fatty acids *via* amide bonds and different head groups. The head groups are comprised of hydrogen (ceramide), phosphocholine (sphingomyelin), and oligosaccharides (glycosphingolipids) (107). Plasmalogens are a species of phospholipids distinguished by having a vinyl ether bond linking a fatty aldehyde to the stereospecific numbered carbon 1 (*sn1)* position and a fatty acyl esterified at the *sn2* position of the glycerol moiety (10, 11, 109). Sphingomyelin (36%) is the most abundant complex lipid in breast milk. It is followed by glycerophospholipids, which are comprised of primarily of phosphatidylethanolamine (29%) and phosphatidylcholine (25%) (53, 55, 110).

### Cholesterol

Free and esterified cholesterol is present in the milk fat globule. The total cholesterol content in human milk is 9–15 mg/ml. Cholesterol plays several functions in infants. It is the building block of myelin in the nervous system, and also acts as a substrate for the synthesis of bile acids, lipoproteins, vitamin D, and hormones (55).

## Maternal factors affecting fatty acid composition in breast milk

The percentage of fatty acids in breast milk is affected by several factors, including maternal diet (53, 111, 112). Studies have found that maternal dietary intake affects SFAs such as capric acid, lauric acid, myristic acid, MUFAs such as oleic acid, and PUFAs such as EPA, DHA, LA, and ALA (112–118). Collectively, these studies show that the diet of lactating mothers is a very potent mediator of breast milk lipid composition during lactation. In addition, maternal age, body mass index, stress, and genetics have all been shown to influence breast milk fatty acid composition (119, 120). Maternal body mass index has been shown to have a positive correlation with concentrations of SFAs and a negative correlation with Omega-3 fatty acids in breast milk (120, 121).

## Functional lipids in breast milk and their role in improving infant health

Functional food can be defined as foods or ingredients which assist in decreasing the risk of disease and/or improving health beyond nutritive purposes (122). Functional lipids are found in a wide variety of food. They are consumed with daily food intake, however, they have physiological benefits reported to reduce the risk of disease (123). Functional lipids in breast milk include MCTs, DAGs, CLAs or CLNs, omega 3, 6, and 9 fatty acids, phospholipids, plasmalogens, sphingolipids, and FAHFAs (11, 53, 124).

### Physiologic role of breast milk functional lipids

Here we review the functional breast milk lipids that have been reported to impact physiologic processes in infants based on human observational studies, clinical studies, and randomized controlled trials. Butyrate (4:0) is a common SFA present in breast milk and acts as an anti-inflammatory and gene expression regulator in the intestine of infants (125). Other SFAs such as caproic (C6:0), caprylic (C8:0), capric (C10:0), and lauric (C12:0) acids inhibit the biological activities of microorganisms (27). C10:0, C12:0, and C14:0 are synthesized in the mammary gland and their presence at the sn1, sn2, and sn3 positions results in the formation of MCTs (126). MCTs are digested without bile salts and are passed along with short chain fatty acids directly to the liver for metabolism without lipoprotein (127). They are beneficial for infants who have abnormalities with fat digestion, absorption, transport, and metabolism (128, 129). Palmitic acid (C16:0) is the major SFA in human milk. It is typically located at the sn2 position of the glycerol moiety of triglycerides. Palmitic acid is converted into sn-2 monoacylglycerol (MAG) by pancreatic lipase at the sn-1 and sn-3 positions (130). Palmitic acid improves fat and calcium absorption, decreases intestinal injury and inflammation by upregulation of antioxidant enzymes, and also plays an important role in mucosal and gut microbiome homeostasis (21). The long-chain PUFAs are the most abundant PUFAs in breast milk. PUFAs play important roles in visual, immune, cognitive, and motor development in infants (131). CLA acts as a growth promoter in infants (132). Phospholipids containing PUFAs act as antioxidants in the gut mucosa (133). Phospholipids are important components of the central nervous system. They play a major role in cell signaling, cell proliferation, maturation of the brain, and reduction of inflammatory diseases in infants (3). They also play a crucial role in the maturation of infant lungs (3–6), which helps to reduce respiratory diseases in the newborn infant (131). PC and sphingomyelin in breast milk release large amounts of choline which plays an important role in neuronal development (134). Choline is a precursor for the neurotransmitter acetylcholine which aids in brain development (55). Choline also helps protect the gastrointestinal tract from infection (3). Plasmalogens maintain the cell membrane physical bilayer properties which is essential for membrane fusion, signal transmission, preventing oxidative stress, and mediate the inflammatory response in infants (11, 109). Sphingomyelin has a critical role in brain and immune development (106). Glycosphingolipids assist in the cognitive development of infants during the first 6 months (55). Table 1 contains a summary of functional breast milk lipids and their physiologic role in infants. However, the role of functional lipids remains largely unexplored in infants and additional studies are needed to further characterize these relationships. Emerging bioinformatics pathways will aid in identifying the relationships between lipid biomarker and clinical outcomes.

**TABLE 1:** The summarization of functional lipids and their role.

Functional lipid	Physiologic role of functional lipid
Short chain fatty acid	• Anti-inflammatory • Antimicrobial
Medium chain fatty acids	• Gastrointestinal development
Monounsaturated fatty acids	• Visual development • Immune development • Cognitive and motor development
Poly unsaturated fatty acids	• Visual development • Immune development • Cognitive and motor development
Conjugated fatty acid	• Growth promoter
Phospholipids	• Antioxidant in the gut mucosa • Brain and neuron development • Lung development • Cell signaling and proliferation • Anti- inflammatory
Sphingolipids	• Neuron development • Gastrointestinal development
Plasmalogens	• Prevent oxidative stress • Anti-inflammatory
Fatty acid esters of hydroxy fatty acids	• Anti-inflammatory • Anti-diabetic properties

## Conclusion

Human breast milk is the recommended source of nutrition for infants in the first 6 months of life and beyond due to the long-term positive impacts of its functional components, including lipids. Lipids not only provide energy, but they also play several additional roles such as aiding in the development of the immune and neurologic systems, as well as regulating metabolism. Functional lipids in breast milk may play a major role in infant outcomes. Characterizing the breast milk lipidome and linking its functional lipid components to infant outcomes will allow for targeted and precise lacto-engineering to optimize the health of children. Therefore, the development of biochemical and bioinformatic pathways to advance breast milk lipidomics may be essential in improving the health of future generations. More attention should be given to breast milk functional lipidome, maternal physiology and transgenerational influences mediating children’s health. Doing this from a systems biology perspective would provide indispensable information to further advance our understanding in this area in the scientific community.

## Author contributions

FE, SS, SC, and RT: conceptualization. MG and SE: writing-original draft. MG, FE, SS, SC, RT, and SE: writing-review and editing. All authors read and approved the final draft of the manuscript.
